# Perception of the Attributes of Sherry Wine and Its Consumption in Young People in the South of Spain

**DOI:** 10.3390/foods9040417

**Published:** 2020-04-02

**Authors:** Serafín J. Cruces-Montes, Ana Merchán-Clavellino, Antonio Romero-Moreno, Alberto Paramio

**Affiliations:** 1 Department of Psychology, Faculty of Education Sciences, University of Cádiz, 11519 Puerto Real, Spain; antoniofrancisco.romero@uca.es (A.R.-M.); alberto.paramio@uca.es (A.P.); 2 INDESS (University Institute for Sustainable Social Development), University of Cádiz, 11406 Jerez de la Frontera, Spain

**Keywords:** consumer behaviour, wine attribute, sherry wine, gender

## Abstract

The wine with the designation of origin “Jerez-Xerez-Sherry” is one of the most produced in Spain and with a greater volume of exports abroad. This study analyzes the preferences of Sherry Wine and its different varieties, based on gender and knowledge and interest in the world of wine. Similarly, the influence of the attributes of Sherry Wine on its choice and consumption is investigated. For this purpose, 1502 participants (1407 wine consumers) were recruited; among the consumers, 58.5% are women, and 74.3% have little knowledge of wine (Mean age 22.6; SD = 3.07; range 18–30). Data collection was done through an online survey. The results reveal that among the types of wines from Sherry, the Fino and the Manzanilla are the most chosen. The young people who have the highest consumption of wine are those who have the most prior knowledge of wine. Also, these young people attribute their choice of wine to intrinsic factors, and even women and connoisseurs are more important in this type of dimension. But the importance of the attributes differs according to the types of wines (Fino/Cream—flavor, Palo Cortado/Pedro Ximénez—color, Amontillado/Manzanilla—price and Oloroso—prizes). These findings will allow establishing measures for their promotion, as well as for the design and specific sales strategies for each type of wine.

## 1. Introduction

The latest data from the International Organization of Vine and Wine (OIV) show that the consumption of wine in 2018 totalled 246 MHL [1]. Of the quantities consumed, Spain was the largest exporter, with statistics representing 19.4% of the global market, that is, with a volume of 20.9 MHL of exports and 34.3 MHL of production [2]. In this way, Spain has the largest land area of vineyards, with a total of 969 MHA [1].

Of wines produced in Spain, Sherry Wine (SW) is more significantly produced in the south of Spain (region of Cádiz), framed in the vast “Sherry Wine Region” vineyards. Weather conditions and a particular production system, such as the dynamic system “soleras” and “criaderas” [3] play a crucial role in the nature of these wines. According to Regulatory Council of Denomination of Origin “Jerez-Xérès-Sherry”, “Manzanilla-Sanlúcar de Barrameda” and “Vinagre de Jerez” (RCDO) in the Sherry Wine Region there are 63 wineries dedicated to the breeding and processing of Sherry Wine, and these wines are the product with the oldest designation of origin in Spain [4].

The SW is based on three types of grape; the “Palomino Fino” grape (the basis of the total production), the “Pedro Ximénez” grape and the “Moscatel” grape [5,6]. These types of grape are used for the production of the so-called “Generosos” wines, characterized by being dry (with a maximum residual sugar quantity of five grams per litre), known as “Manzanilla”, “Fino”, “Amontillado”, “Oloroso” and “Palo Cortado”. Generosos Liqueur Sherry Wines, such as “Pale Cream”, “Medium” and “Cream”, are obtained based on the type of “generosos” wine used as a base, and the final levels of sweetness of the blend (always with a sugar content of over five grams per litre). Finally, we can find naturally sweet Sherry Wines, produced according to the variety of grape used: “Moscatel” and “Pedro Ximénez” [4].

In general, 81.3% of the Spanish population between the age of 15 and 64 has consumed alcohol in the last year, with wine being the second most popluar choice, with a 26.1% preference over other alcoholic beverages such as beer, tall drinks, strong liquors, vermouth, and fruit liqueur, according to the latest data of 2019 [7]. However, if we focus on ages between 18 and 30 years, the consumption of alcohol in the last year increased by almost ten percent, even though, this increase is not observed in the preference for wine [7]. However, in 2019, the Spanish Wine Market Observatory (OEMV) [8] reported that wine consumption grew by 7.2% in Spain, due to changes in wine distribution and a wine tourism boom.

For this reason, studies on the factors that determine the purchase and choice of wine have increased, especially those that analyze the influence of consumer attitudes and the relevance of consumption contexts. However, few studies have addressed the incidence of the characteristics of the wine, both intrinsic (taste, aroma, graduation, etc.) and extrinsic (brand, labelling, bottling, price, etc.), but we can even find fewer studies that focused their population study on the youth segment [9]. Thus, as Silva, Figueiredo, Hogg, and Sottomayor [10] suggested, research has focused primarily on quantitative studies on trends in wine consumption in adults, and when they have focused on the youth group, they have only focused on issues related to consumer abuse [11,12,13,14]. Nevertheless, some knowledge of the elements that motivate the non-abusive consumption of wine among young people will help wine producers have a better idea of how future consumers will react, as well as to better understand their expectations [15].

In this sense, research on the preferences of young people for the attributes of wine has concluded that characteristics such as price, taste and brand value are reasons related to their choice [16,17,18]. In the case of young people, the price of wine is one of the main reasons that can lead to the choice of cheaper alcoholic beverages [19,20,21], and the taste of wine is frequently indicated as the main reason for aversion among young non-consumers [10]. Therefore, as Capitello, Agnoli, and Begalli [22] pointed out, when it comes to reaching young consumers, marketing approaches should be specially adapted to them, promoting communication for educational purposes, acknowledging intrinsic and sensorial signals of wine, such as aroma and flavor, among others. Promoting knowledge and interest in the world of wine has proven effective in adults, increasing the amount of wine purchases [23,24].

Similarly, aesthetic aspects, such as packaging and labelling, are also important attributes that explain the choice of this product among young people [10,25]. Elliot and Barth [26] found that the wine choices that young people make (defined as Millennials by the authors) are fundamentally influenced by extrinsic type factors (bottling, labelling, and design) rather than by the characteristics of the product itself (producer, country of origin, type of wine, graduation, quality or solera). But as young people get older and gain experience drinking wine, their opinions and references regarding extrinsic factors may change to intrinsic factors. In addition, they express a desire to develop skills and knowledge about wine in the future, which denotes a purpose of wanting to learn about, understand and appreciate wine for its sensory qualities [10]. Thus, knowing the reasons of consumption would facilitate the realization of specific promotions to better target different age groups and different genders. As of today, it is not clear whether men and women drink wine for the same reasons [27]. In this sense, women seem to make less abusive use of wine than men and are more sensitive to sustainable wine, even among millennials [14,28,29,30].

In short, young consumers evaluate wine as a multifaceted category [31], so that, due to the complexity of this product, novice consumers often find it difficult to appreciate its quality, which can affect the decision and choice processes [32,33]. Young people, although they have limited experience, can assess wines using specific characteristics such as origin, harvest, label, brand and grape variety [34,35]. Thus, as previously mentioned, it would be desirable to promote greater dissemination and publicity of the wine addressed to this group, using aspects such as attention to value for money, flavor, innovative packaging and environmental emphasis [18], among others. In transmitting such information, it would be interesting to use internet-based channels and social networks, since they are sources mainly used by young people [36]. By always encouraging responsible consumption, it will allow us to appreciate wine as an important nutritional, cultural and economic elements, which can be beneficial for health when consumed in moderation [37,38]. In summary, as Tealgle, Mueller, and Lockshin [39] indicate, although young millennials are certainly neophytes in terms of participation and subjective knowledge of wine, they are learning quickly.

Therefore, the present study focuses on analyzing the current behaviour of the Sherry Wine consumer, and its varieties, in a large sample of young people between 18 and 30 years old. It is intended to investigate the importance attached to the different attributes of Sherry Wine, and especially to its types of wine. Also, taking into account the current interest in the segmentation of the wine consumer, the exploration is proposed according to demographic aspects such as gender and behaviour, based on knowledge and interest in the world of wine. In conclusion, knowing the consumer profile of this type of wine will help Spanish wineries to direct commercial aspects, by developing and adapting their wines to the new behaviours of these young users.

## 2. Materials and Methods

The sample consisted of 1502 participants, community members of the University of Cádiz (94.2% undergraduate and master students, teaching and administrative staff). Of all those who participated in the study, 1407 consumed SW at least once a year. Among the consumers, 58.5% are women and 74.3% are considered to have little knowledge regarding wine (mean age 22.6; SD = 3.07; range 18–30). These participants were divided to analyze the differences in the consumption and the perception of the importance of wine attributes by gender and by knowledge and interest in the Sherry Wine. Participation in this study was voluntary and confidential. The study was conducted in compliance with the Declaration of Helsinki of 1975, and all participants signed the informed consent. All students completed the online self-report questionnaires and the criteria for joining the analysis of this study was to be in the 18–30 age range.

The authors prepared a survey. The survey was sent to the community of the University of Cádiz through professional emails. The survey was divided into three parts: data of gender and knowledge and interest about the SW, frequency of SW consumption and importance of intrinsic and extrinsic attributes. The intrinsic attributes were composed of Flavor, Aroma, Color, Alcoholic Content and Ecological characteristics; and the extrinsic attributes by Price, Labels, Prizes, Brand and Bottling.

Frequencies and basic descriptive statistics of the questionnaire variables were analyzed. As the principle of normality in the variables was not fulfilled, non-parametric analyses of independent samples were carried out to verify if there were significant differences between the groups (*U* Mann-Whitney). In addition, multiple linear regressions were carried out to develop the predictive models of consumption in each type of wine.

Data analysis and processing were performed with the IBM Statistical Package for the Social Sciences (SPSS) v25 software (IBM Corporation, Armonk, NY, USA). The results were considered significant with a *p* < 0.05.

## 3. Results

The results on the sample of 1502 young adults show a relatively low-sporadic consumption in general. In total, 17.4% of the participants declared that they only consume sherry once a year and 33.2% consume wine once every three months. Daily consumers make up only 2.4% of the total sample, and 6.3% said they never drink Sherry Wines. Table 1 shows the frequencies of consumption according to the types of wines of “The Sherry Wine Region”.

Next, in Table 2, the descriptive statistics of Sherry Wine consumption are presented, both for the total sample and the segmentation variables of the study, such as gender and knowledge and interest in the world of wine.

Statistical analyses showed no significant differences between men and women regarding the consumption of wine, neither for consumption in general nor in its different types. However, we find differences between those who have a previous interest and knowledge about the world of Sherry Wine and those who do not, both for consumption in general and for different types (see Table 2).

**Figure 1:**
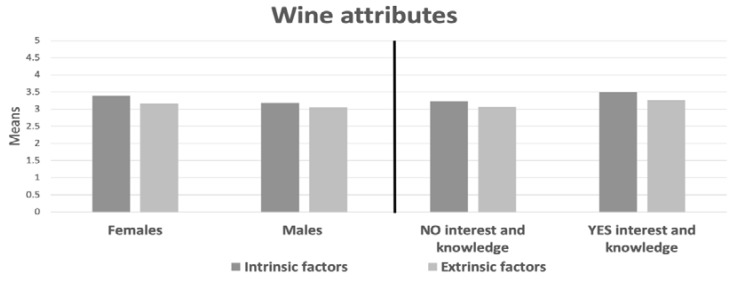
Mean of importance intrinsic and extrinsic attributes, genders, knowledge, and interest in the world of wine.

Regarding the importance of intrinsic and extrinsic factors when choosing Sherry Wine in consumers, significant differences are observed (Z = −10.052; *p* = 0.000), so that inherent factors are more important than extrinsic (see Figure 1).

The same applies to each gender (Men: Z = −4.905; *p* = 0.000 and Women: Z = −8.978; *p* = 0.000) and those who have knowledge or not about the world of wine (Yes knowledge Z = −5.839; *p* = 0.000 and No knowledge: Z = −8.179; *p* = 0.000). There are also differences between genders in the different factors, assessing both types of attributes with a higher level of importance for women compared to men (Intrinsic: *U* = 199768.5; *p* = 0.000 and Extrinsic: *U* = 220930.5; *p* = 0.010). On the other hand, the values increase significantly in both factors for drinkers who have previous knowledge with respect to those who do not have any (Intrinsic: *U* = 154773.5; *p* = 0.000 and Extrinsic: *U* = 164854; *p* = 0.010).

Of all the attributes selected for analysis, described in Table 3, Flavor, Aroma, Alcoholic Content, Ecological Characterisitcs, Price and Bottling showed significant differences concerning gender (p <.05), with women granting greater importance to these attributes than men. However, concerning prior knowledge, except for the Ecological attribute, significant differences appear in the level of importance related to the attributes, reflecting a higher level of importance on the part of the participants with prior knowledge and interest in wine.

Various regression models were made on the general consumption of Sherry and for each type, using the input variables: gender, knowledge and interest in wine, importance for taste, for aroma, for color, for alcoholic content, for its ecological character, for the price, for its label, for its prizes, for the brand and bottling. These models are shown in Table 4. In the table, we can see the attributes that significantly predict the consumption of wine in general, and in each of its types.

## 4. Discussion

This study tries to analyze in a sample of young people the current behavior of the SW consumer, and its varieties. In general, the results confirm that 93.7% of the proportion consumes SW, thereby corroborating the data in the Spanish population, where young people drink alcohol with percentages close to 90% [7]. The latest surveys [7] also informed us that wine is the second drink of choice, with a 26.1% preference over other beverages. Although we cannot confirm this option, we observe that the frequency of consumption is not very high, as the young population’s daily consumption only reaches 2.4%. Moreover, wine is a drink only chosen sporadically, as the highest percentage of consumption is once every three months among young people.

**Figure 2:**
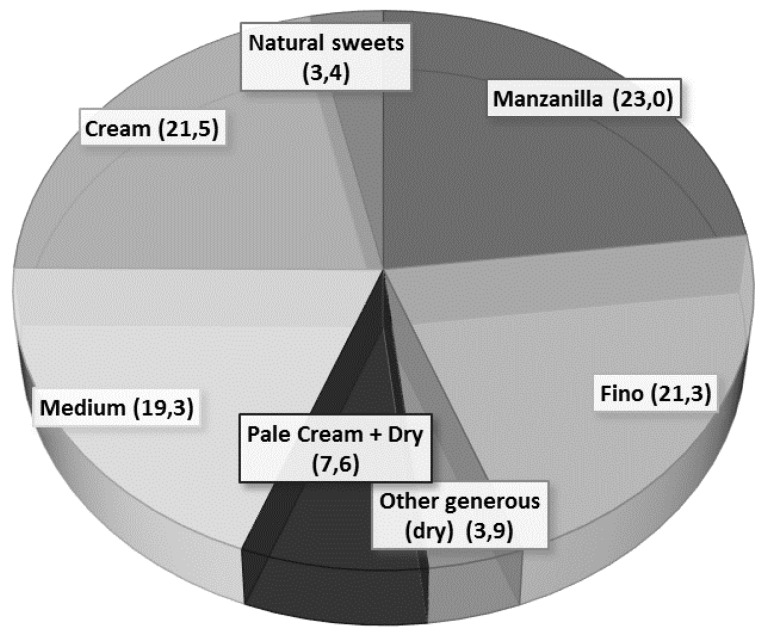
Percentage of sales of Sherry Wine, by types [40].

Regarding the consumption of the SW variety, our data show that, in order of preference, they choose Fino and Manzanilla, with very similar consumption percentages (77.96% and 76.83%, respectively). The data also confirm that Manzanilla is the best-selling wine, with 23% of the total, followed by Fino with 21.3% [40]. However, although sales of Pedro Ximénez or sweet wines represent the lowest percentages according to the Regulatory Council (see Figure 2) [40], we observe that it is the third most consumed type of wine. This disagreement may be due to the increase in sales that has been found since 2018 or due to the fact that the Spanish prefer to buy Pedro Ximénez over other wines [40]. Ultimately, the Amontillado, the Oloroso and the Palo Cortado barely represent 4% of total sales, confirming that they are the least consumed wines in the young population.

In general, few studies have shown trends or distribution of wine consumption in young people, and even less, of SW. When they have, they have dealt with issues related to consumer abuse [11,12,13,14]. It is not yet clearly established whether men and women drink wine on the same occasions [27]. This situation turned out to repeat itself in our population since we do not find significant differences between genders in wine consumption. On the other hand, other studies have shown that men drink slightly more wine than women and these differences worsen after 35 years [29,30,31,32,33,34,35,36,37,38,39,40,41]. We do not find such differences in our study as our population is between 18 and 30 years, and those differences in consumption are not yet observed. Also, these differences are reversed depending on the type of wine, so that men drink more red wines and women consume white wine [42]. It has also been found that these slight differences between gender diminish with the growing experience in wine [42]. In this way, it is observed how knowledge and prior interest in the world of wine plays an essential role in its consumption. Our results show that people who have more experience with wine consume more Sherry Wine, in all its varieties. Purchase data form other studies corroborate that those who visited wineries helped increase the purchase percentages by 13% [23,24].

As a result of the multifaceted and complex nature that young people attribute to the choice of wine [31,43], it was necessary to increase knowledge about the importance given to the different attributes for the consumption of SW, and especially for their types. Also, with the current interest in defining consumer segmentation, demographic aspects such as gender and those based on knowledge and interest in the world of wine were investigated. Thus, some studies have shown how young people’s wine choices are fundamentally influenced by extrinsic type factors (bottling, labelling, and design) rather than by the characteristics of the product itself (producer, country of origin, type of wine, graduation, quality or solera) [15,26]. Even women give greater importance to the external signals of the wine than to the internal ones, according to Bruwer and McCutcheon [42].

However, our results present a preference for attributing their choices of Sherry Wine consumption more to intrinsic or product factors than to extrinsic factors. Therefore, these data are not consistent with the previous literature. To explain this, we must take into account that the study population is from a wine region and according to specific authors we could consider them as quality consumers; that is, consumers with high involvement and great reflection in their decision process. In summary, they need more senior level signals for their choice of purchase, such as intrinsic factors [43,44,45,46].

Initially, we expected to find these differences and this trend in women and those with higher prior knowledge, since women have more capacity to capture the subtleties and smells of wine [47] and because the so-called “connoisseurs” need more information; that is to say, to search information before its purchase, using the opinion of the experts by reading wine magazines and books. In short, they are more involved in the decision process before making a purchase, and will, therefore, be guided by higher marks [44,45,46,48]. Concerning the search for information, Millennials have demonstrated active participation in activities related to wine, such as the search for emotional, experimental and educational value [15].

Next, we considered investigating which attributes between intrinsic and extrinsic have higher predictive power and show significant associations with the different types of SW. In this way, our results will help Spanish wineries to direct the commercial aspects, by developing and adapting their wines to the most outstanding elements in the population. 

After reviewing the different regression models, both gender and etiquette are unimportant variables when predicting the consumption of SW, so we are in line with several studies that demonstrate this low importance [42,46]. However, being knowledgeable or having experiences related to wine is a good predictor for the consumption of SW, although observing varieties, both for Cream, for the array of Palo Cortado and Pedro Ximénez do not present a strong association as in the rest of wines. Based on these results, it would be interesting to investigate whether the amounts of sugar can explain the trend detected. While, in general, our data are in line with other studies, as mentioned above, prior knowledge and interest in wine predict its consumption and purchase intention [23,24].

It should be noted that young people perceive specific characteristics such as aroma, flavor, price, brand, color, and prizes as essential factors for their consumption of SW, and it is in line with the most chosen attributes in different types of populations. For example, taste and smell is an indicator of “commitment” to the product, price is the best attribute in younger consumers, and the brand is another important factor because it relates to the consumer expectations that the associated images transmit in advertising [9,34,35,46,49]. Next, regarding the types of wine, it has been found that Fino and Cream are mostly consumed for flavor, Palo Cortado and Pedro Ximénez are mostly consumed for their color, Amontillado and Manzanilla for their price and finally Oloroso is more consumed for the prizes received.

Notice that the models described predict 19.6% of the consumption of SW, and that the percentages of the models with the analyzed variables vary from 7.1% of the predictive power for the consumption of Pedro Ximénez up to 10% of Fino consumers, contributions with values to highlight and that may have a great interest in the wine sector. In addition, taking into account the fact that young consumers evaluate wine according to a multitude of factors [31], for example, the newer ones have more difficulty in knowing its quality and that it may affect their choices [32,33]. 

With these results, Spanish wineries can be helped to direct their efforts toward certain commercial aspects, by developing and adapting their wines to the new behaviors of these young consumers, while creating a specialized marketing campaign for the variety of wines presented by the sherry frame. We believe that this study places value and places its focus on the development of wine tourism and its landscapes worldwide and that it is of vital importance for the 2020-2024 Strategic Plan, between the OIV and the World Tourism Organization, since it has been shown that boosting wine and wine distribution increases wine consumption [8]. The limitations of this study open opportunities for future research; for example, more extensive and random samples, adding additional questions to the current survey and incorporating more detailed information on psychosocial aspects.

## 5. Conclusions

The results of this preliminary study suggest that there is a relationship between prior knowledge of and interest in wine culture and wine consumption in young adults. Despite being very focused in the wine region of Jerez, this research provided us with a way to delve into the psychosocial aspects of a product whose complexity and variety is further of the organoleptic and chemical elements, conventional approaches in wine research. Considering the limitations, our results show that wine consumption by young people is low, and consumers’ gender is not a determining factor in consumption and the perceptions of its intrinsic and extrinsic attributes. Even so, the consumption of wine is recognized to different dimensions: Fino and Cream for its flavor, Palo Cortado and Pedro Ximénez for its color, Amontillado and the Manzanilla for its price and finally Oloroso are more consumed by the awards received. For future research, we recommend a more extensive and random sample, adding additional questions to the current survey and incorporating more detailed information on psychosocial aspects. Emphasizing knowledge of the culture of wine and involving local wineries of the denomination of origin area, since the awareness of these aspects is lower, will probably achieve a better product promotion and better specific marketing strategies, attending to the most important factors linked to each of the types of wine.

## Figures and Tables

**Table 1:** Frequencies of consumption of the types of Sherry Wines.

Consumption	Fino		Manzanilla		Oloroso		Cream		Amontillado		Palo Cortado		Pedro Ximénez	
	*n*	%	*n*	%	*n*	%	*n*	%	*n*	%	*n*	%	*n*	%
Never	331	22	348	23.2	591	39.3	701	46.7	833	55.5	971	64.6	361	24.0
Once a year	513	34.2	544	36.2	356	23.7	300	20.0	306	20.4	261	17.4	440	29.3
Once every three months	351	23.4	374	24.9	316	21.0	259	17.2	202	13.4	163	10.9	368	24.5
Once a month	203	13.5	163	10.9	170	11.3	136	9.1	109	7.3	73	4.9	226	15.0
Once a week	90	6.0	64	4.3	63	4.2	94	6.3	48	3.2	29	1.9	93	6.2
Daily	14	0.9	9	0.6	6	0.4	12	0.8	4	0.3	5	0.3	14	0.9

**Table 2:** Descriptive analyzes (means and standard deviations) and statistics (Mann–Whitney *U* Test) of Sherry Wine consumption for the general sample, between genders and between knowledge and interest in the world of wine.

	*n*	Overall		Gender				*U*	Knowledge and Interest				*U*	
				Female		Male			No		Yes			
		M	SD	M	SD	M	SD		M	SD	M	SD		
Sherry Wines	1407	2.51	1.07	2.49	1.06	2.53	1.08	234956.5	2.35	1.02	2.94	1.08	131864.5	**
Fino	1171	1.92	1.01	1.92	1.03	1.94	0.99	164103.5	1.8	0.95	2.25	1.1	105501.0	**
Manzanilla	1154	1.8	0.93	1.78	0.92	1.84	0.95	158175.5	1.69	0.85	2.11	1.07	101954.0	**
Oloroso	911	1.95	0.96	1.95	0.97	1.96	0.94	101150.5	1.82	0.9	2.26	1.01	66486.0	**
Cream	801	2.07	1.07	2.09	1.05	2.05	1.09	75458.5	1.98	1.01	2.25	1.16	62819.0	**
Amontillado	669	1.87	0.97	1.89	0.96	1.84	0.99	53444.5	1.72	0.86	2.13	1.1	41109.5	**
Palo Cortado	531	1.78	0.94	1.78	0.9	1.79	0.99	34768.0	1.68	0.87	1.95	1.03	28893.0	**
Pedro Ximénez	1141	2.01	1.01	2	1	2.03	1.03	155901.5	1.92	0.98	2.26	1.06	105030.0	**

Notes: ** *p* < 0.001; SD, Standard Deviation; M, Mean.

**Table 3:** Descriptive analyzes (means and standard deviations) and statistics (Mann–Whitney *U* Test) of the perception of the importance of wine attributes for the general sample, between genders and between knowledge and interest in the world of wine.

Atttribute	Knowledge and Interest				U		Gender				U		Overall	
	No		Yes				Female		Male					
	M	SD	M	SD			M	SD	M	SD			M	SD
Flavor	4.35	0.99	4.51	0.83	227941.5	**	4.44	0.93	4.33	1	287122.5		4.4	0.96
Aroma	3.62	1.09	4.03	0.94	258008.5	**	3.79	1.06	3.62	1.07	294134.5	**	3.72	1.07
Color	2.82	1.16	3.16	1.13	245216	**	2.94	1.15	2.85	1.16	279168.5		2.9	1.16
Alcohol Content	2.76	1.18	2.92	1.12	226350	**	2.93	1.17	2.62	1.13	305622	**	2.8	1.17
Ecological	2.67	1.26	2.82	1.27	223515.5	*	2.84	1.26	2.52	1.23	306622.5	**	2.71	1.26
Price	3.66	1.19	3.59	1.1	196607.5		3.71	1.15	3.55	1.18	295356.5	**	3.65	1.17
Labels	3.1	1.29	3.43	1.2	241097.5	**	3.21	1.28	3.14	1.28	281317		3.18	1.28
Prizes	2.47	1.18	2.81	1.2	244473	**	2.56	1.19	2.56	1.21	271509.5		2.56	1.2
Brand	3.01	1.22	3.24	1.15	233318.	**	3.11	1.2	3.01	1.21	283031.5		3.07	1.21
Bottling	3.03	1.25	3.25	1.13	231961.5	**	3.18	1.22	2.96	1.21	294728.5	**	3.09	1.22

Note: ** *p* < 0.001; * *p* < 0.05.

**Table 4:** Multiple linear regression analysis that predicts variables of the Sherry Wine consumption due to the importance of its attributes.

	*R* ^2^	*F*	Predictor Variables	*Beta*	*t*
Sherry Wine	0.196	29.510 **	Flavor	0.165	4.832 **
			Aroma	0.178	5.231 **
			Color	0.091	3.066 **
			Price	−0.136	−4.599 **
			Prizes	0.068	2.112 *
			Brand	0.095	3.062 **
			Knowledge and interest	0.169	6.887 **
Fino	0.107	12.71 **	Flavor	0.148	3.779 **
			Color	0.082	2.403 *
			Price	−0.100	−2.971 **
			Prizes	0.103	2.721 **
			Brand	0.070	1.967 *
			Knowledge and interest	0.143	5.055 **
Manzanilla	0.085	9.98 **	Aroma	0.124	3.121 **
			Price	−0.124	−3.531 **
			Brand	0.082	2.245 *
			Knowledge and interest	0.146	5.055 **
Amontillado	0.097	6.99 **	Flavor	0.150	2.916 **
			Color	0.138	2.972 **
			Price	−0.151	−3.504 **
			Bottling	−0.100	−2.181 *
			Knowledge and interest	0.168	4.487 **
Oloroso	0.096	9.061 **	Flavor	0.101	2.213 *
			Aroma	0.109	2.385 *
			Price	−0.115	−3.006 **
			Premios	0.110	2.592 *
			Knowledge and interest	0.166	5.116 **
Cream	0.083	6.997 **	Flavor	0.202	4.276 **
			Color	0.156	3.592 **
			Price	−0.135	−3.341 **
			Brand	0.110	2.501 *
			Knowledge and interest	0.078	2.238 *
Palo cortado	0.069	4.266 **	Color	0.140	2.655 **
			Price	−0.130	−2.635 **
			Prizes	0.119	2.029 *
			Knowledge and interest	0.101	2.362 *
Pedro Ximénez	0.071	8.3 **	Color	0.128	3.628 **
			Price	−0.091	−2.596 *
			Prizes	0.103	2.670 **
			Knowledge and interest	0.110	3.769 **

Note: ** *p* < 0.001; * *p* < 0.05.
